# Therapeutic potential of 4-phenylbutyric acid against methylmercury-induced neuronal cell death in mice

**DOI:** 10.1007/s00204-024-03902-3

**Published:** 2024-10-27

**Authors:** Ryohei Miki, Ryosuke Nomura, Yuta Iijima, Sho Kubota, Nobumasa Takasugi, Takao Iwawaki, Masatake Fujimura, Takashi Uehara

**Affiliations:** 1 Department of Medicinal Pharmacology, Graduate School of Medicine, Dentistry and Pharmaceutical Sciences, Okayama University, Okayama, 700‑8530 Japan; 2 Division of Cell Medicine, Department of Life Science, Medical Research Institute, Kanazawa Medical University, Ishikawa, 920-0293 Japan; 3 Department of International Affairs and Research, National Institute for Minamata Disease, Kumamoto, 867‑0008 Japan

**Keywords:** Methylmercury, Neuronal cell death, Endoplasmic reticulum stress, Unfolded protein response

## Abstract

Methylmercury (MeHg) is an environmental neurotoxin that induces damage to the central nervous system and is the causative agent in Minamata disease. The mechanisms underlying MeHg neurotoxicity remain largely unknown, and there is a need for effective therapeutic agents, such as those that target MeHg-induced endoplasmic reticulum (ER) stress and the unfolded protein response (UPR), which is activated as a defense mechanism. We investigated whether intraperitoneal administration of the chemical chaperone, 4-phenylbutyric acid (4-PBA), at 120 mg/kg/day can alleviate neurotoxicity in the brains of mice administered 50 ppm MeHg in drinking water for 5 weeks. 4-PBA significantly reduced MeHg-induced ER stress, neuronal apoptosis, and neurological symptoms. Furthermore, 4-PBA was effective even when administered 2 weeks after the initiation of exposure to 30 ppm MeHg in drinking water. Our results strongly indicate that ER stress and the UPR are key processes involved in MeHg toxicity, and that 4-PBA is a novel therapeutic candidate for MeHg-induced neurotoxicity.

**Supplementary Information:**

The online version contains supplementary material available at 10.1007/s00204-024-03902-3.

## Introduction

Mercury is a highly toxic environmental substance and is listed by the World Health Organization (WHO) among the top 10 chemicals of public health concern. Methylmercury (MeHg), which has garnered significant attention, is generated when inorganic mercury released into the atmosphere by volcanic eruptions and gold mining is deposited into the ocean and converted by microorganisms. (Compeau and Bartha 1985; King et al. 2000). MeHg accumulates in the aquatic food chain through bioaccumulation, and the ingestion of fish, especially tuna, is a major source of MeHg exposure in humans (Mahaffey et al. 2004). MeHg easily crosses the blood–brain barrier and exhibits neurotoxicity, as exemplified by Minamata disease, in which the cerebellum and cerebral cortex are injured (Eto and Takeuchi 1978; Kerper et al. 1992). The mechanism of MeHg toxicity remains unclear, and a useful therapeutic agent against MeHg toxicity has not been identified.

MeHg is electrophilic, covalently binds to cysteine thiol groups, which are nucleophilic substituents of proteins (*S*-mercuration), and induces changes in the activity of modified proteins (Clarkson 1972). We previously found that protein disulfide isomerase (PDI), an enzyme responsible for protein folding, is a target of *S*-mercuration, and that endoplasmic reticulum (ER) stress (accumulation of abnormal proteins in the ER) is induced through enzyme inactivation (Makino et al. 2015; Usuki et al. 2008).

To avoid the damage caused by ER stress, cells activate three stress sensor proteins, IRE1α (inositol-requiring enzyme 1α), PERK (PKR-like endoplasmic reticulum kinase (PERK), and ATF6 (activating transcription factor 6 (ATF6) (Walter and Ron 2011). Our previous in vitro studies revealed that MeHg activates the PERK pathway, which is a mediator of ER stress, and, at high concentrations, inhibits the IRE1α-XBP1 (X-box binding protein) pathway, which functions in cell survival (Hiraoka et al. 2017). Furthermore, in mouse brains exposed to MeHg in drinking water, MeHg induced a neuron-specific increase in ER stress, expression of the downstream apoptosis-promoting factor, CHOP (C/EBP homologous protein), and apoptotic neuronal cell death (Nomura et al. 2022). These results indicate the involvement of ER stress/UPR signaling in MeHg-induced neuropathy and its potential as a therapeutic target.

To test these hypotheses, we examined whether 4-phenylbutyric acid (4-PBA), a chemical chaperone, ameliorates central nervous system injury in ER stress-activated indicator (ERAI)-Venus Tg mice treated with MeHg in drinking water, an animal model of MeHg neurotoxicity. ERAI can detect the activation of the IRE1-XBP1 pathway during ER stress by detecting the expression of Venus due to a frameshift caused by XBP1 splicing. Venus staining can also be used to analyze the spatiotemporal expression of XBP1s, which is difficult to detect. Previous studies have shown that the sensitivity of ERAI mice to MeHg is similar to that of WT mice (Hiraoka et al. 2021). We also showed that MeHg administration induced the expression of the PERK pathway and CHOP, which is downstream of the PERK and ATF6 pathways (Nomura et al. 2022), following the expression of the ERAI signal, suggesting that ERAI mice are an excellent model for analyzing ER stress toxicity caused by MeHg. Chemical chaperones are compounds that are involved in the formation and stabilization of protein structures. 4-PBA reduced ER stress by inhibiting protein aggregation (Kubota et al. 2006). Furthermore, 4-PBA penetrates the blood–brain barrier (BBB), and has significant neuroprotective effects in mouse models of neurodegenerative diseases, such as Alzheimer's disease (AD) and Parkinson’s disease (PD) (Inden et al. 2007; Ricobaraza et al. 2009). Therefore, we administered 4-PBA to mice and examined whether it could inhibit the induction of ER stress by MeHg, resulting in neuronal cell death. MeHg toxicity induces progressive neurodegeneration, making early therapeutic intervention important. In this study, we identified the period at which irreversible damage occurs, to obtain clues to determine the therapeutic time window.

## Materials and methods

### Animals

Male ERAI-Venus mice were generated as previously described (Iwawaki et al. 2004) and maintained on a C57BL/6 background. Male C57BL6N/Jc1 mice were purchased from CLEA Japan (Tokyo, Japan). All mice were housed at the National Institute for Minamata Disease. All mice were housed in plastic cages (three animals per cage) and allowed free access to food (CE-2; CLEA Japan) and water. The animal facility was maintained at 25 ℃ ± 2 °C with a relative humidity of 65% ± 5% under a 12-h light/dark cycle. Mice were euthanized by cardiac blood sampling under deep anesthesia with isoflurane and transcranial perfusion with saline. All authors complied with the ARRIVE guidelines. All animal procedures were performed in accordance with the Guide for the Care and Use of Laboratory Animals issued by the National Institute for Minamata Disease and were approved by the Animal Ethics and Management Committee of the National Institute for Minamata Disease (No. 050912 and 051221).

### MeHg administration

The ERAI-Venus mice were randomly divided into control (n = 6) and 4-PBA-administrated groups (n = 5–6 mice/group). All mice were exposed to MeHg via drinking water containing 50 ppm MeHg (Tokyo Chemical Industry, Tokyo, Japan) as a MeHg-GSH (FUJIFILM Wako Pure Chemical, Osaka, Japan) (1:1) complex, as previously described (Fujimura et al. 2009). To investigate the therapeutic effects of post-treatment with 4-PBA, wild-type (WT) mice were randomly divided into a control group (n = 12) and a 4-PBA-treated group (n = 12 mice/group). All mice were exposed to MeHg in drinking water containing 30 ppm MeHg as the MeHg-GSH (1:1) complex.

### 4-PBA administration

The 4-PBA solution was prepared using equimolar amounts of 4-phenylbutyric acid (Tokyo Chemical Industry) and sodium hydroxide (Nacalai Tesque, Kyoto, Japan) at pH 7.4. For in vivo experiments, mice were intraperitoneally injected with 120 mg/kg/day 4-PBA or saline as a corresponding vehicle control.

### Hindlimb extension analysis

To examine hindlimb impairment induced by MeHg, mice were gently removed from their home cage and suspended by the tail for 10 s. This was performed once a week during the 35 or 56 days of MeHg exposure and the hindlimb extension exhibited by the mice was scored as follows: normal escape extension = 0, incomplete splay and loss of mobility =  − 1, no splay and loss of mobility =  − 2, complete crossing of the hindlimbs =  − 3.

### Preparation of tissue sections

Mice were euthanized by trans-cardiac saline perfusion and the left brain was removed and fixed in 4% paraformaldehyde in 0.1 M phosphate buffer immediately after dissection (Fujifilm Wako Pure Chemicals). The tissue was embedded in paraffin and 5-µm-thick sagittal sections were prepared using a microtome and mounted on glass slides. Before staining, the tissue sections were deparaffinized with xylene and rehydrated with a graded series of ethanol solutions.

### Measurement of mercury content

Mercury deposition was measured as previously described (Hiraoka et al. 2021). ERAI-Venus mice were euthanized at the indicated times, and the cerebral cortex and striatum were removed from their right brains. The tissues were dissolved in 5 N NaOH (Nacalai Tesque, Kyoto, Japan) solution and boiled at 70 °C for 30 min. After neutralization with 5 N HCl (Nacalai Tesque), the total concentration of mercury was measured by the oxygen combustion-gold amalgamation method using an MA2000 analyzer (Nippon Instruments Corporation, Tokyo, Japan), as previously described (Hiraoka et al. 2021).

### TUNEL staining

Apoptosis-induced cell death was monitored by deoxynucleotidyl transferase-mediated dUTP nick-end labeling (TUNEL) assays using the In Situ Cell Death Detection Kit, TMR red (#12,156,792,910; Roche, Basel, Switzerland), according to the manufacturer’s instructions. Briefly, deparaffinized sections were permeabilized with 20 µg/mL Proteinase K (Qiagen, Venlo, Netherlands) in 10 mM Tris–HCl (pH 7.5) for 20 min at 37 °C. After washing with phosphate-buffered saline (PBS), the sections were incubated with the TUNEL reaction mixture for 1 h at 37 °C, washed with PBS, and permeabilized with VECTASHIELD Vibrance Antifade Mounting Medium containing 4ʹ,6-diamidino-2-phenylindole (DAPI) (Vector Laboratories, Burlingame, CA, USA). All images were captured and analyzed using an ECLIPSE Ti confocal microscope (Nikon Instruments, Tokyo, Japan) and NIS-Elements AR imaging software version 4.00.06 (Nikon Instruments). TUNEL-positive cells were identified by the TMR red signal in nuclei stained with DAPI as previously reported (Iijima et al. 2024b).

### Immunofluorescence

Deparaffinized sections were boiled in 10 mM citrate buffer (pH 6) (Genemed Biotechnologies) for 20 min for antigen retrieval prior to antigen detection using a M.O.M. immunodetection kit (#FMK-2201, Vector Laboratories). Immunofluorescence staining was performed overnight at 4 °C using primary antibodies that were diluted in PBS containing 5% BSA. After washing once with PBS, goat anti-rabbit IgG Alexa Fluor 555 secondary antibody (1:200; #A-21428, Thermo Fisher Scientific) or goat anti-rabbit IgG Alexa Fluor 594 secondary antibody (1:200; #A-11012, Thermo Fisher Scientific, MA, USA) was added and incubated for 1 h at room temperature. Nuclei were stained with a mounting medium containing DAPI (Thermo Fisher Scientific). All images were captured and analyzed using an ECLIPSE Ti confocal microscope (Nikon Instruments) and NIS-Elements AR imaging software version 4.00.06 (Nikon Instruments).

## Statistical analysis

Quantitative data are presented as the mean ± standard error of the mean (s.e.m.). Statistical analysis was performed using GraphPad Prism software version 10.0.2 (GraphPad Software, San Diego, CA, USA). Differences between two means were analyzed using a two-way analysis of variance (ANOVA), followed by Bonferroni’s post hoc test. Statistical significance was set at *p* < 0.05.

## Results

### 4-PBA suppresses MeHg-induced ER stress in ERAI mice

**Fig. 1:**
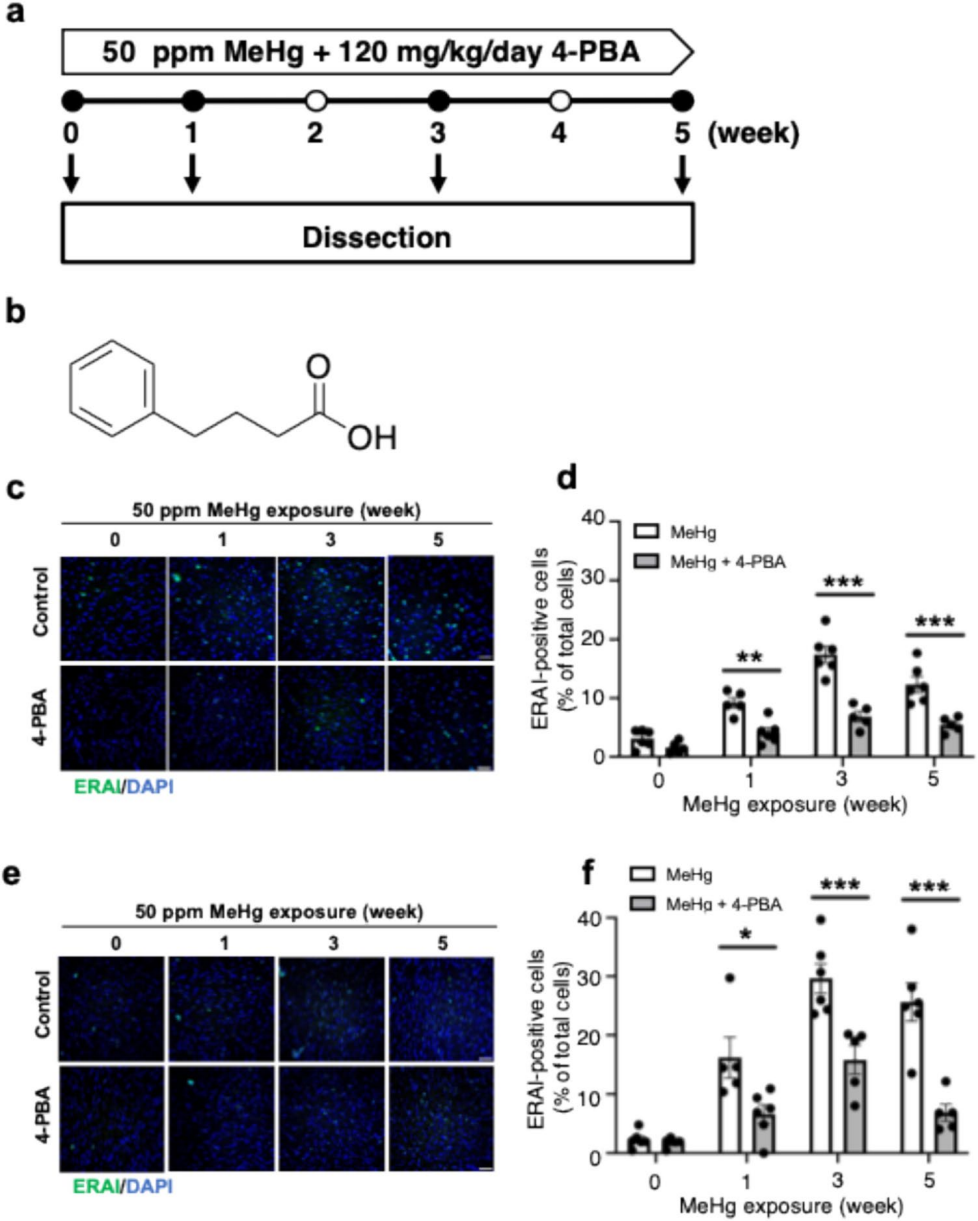
Time course analysis of MeHg-induced ER stress using ERAI-transgenic mice **a** Experimental timeline of subacute exposure to MeHg and 4-PBA. ERAI-transgenic mice were exposed to 50 ppm MeHg in drinking water for up to 5 weeks. In addition, the mice were intraperitoneally inoculated with 4-PBA (120 mg/kg daily). Saline was injected into control animals as a vehicle control. Mice were euthanized at the indicated times and brain tissues were analyzed by immunostaining. **b** Chemical structure of 4-phenylbutyric acid (4-PBA). Representative immunostaining of ERAI in the somatosensory cortex (**c**) and striatum (**e**) of ERAI-transgenic mice exposed to MeHg for the indicated times. The scale bar represents 50 μm. **d**, **f** Quantification of ERAI-positive cells shown in (**c**, **e**, respectively). Data are presented as the mean ± s.e.m. (n = 5–6, *p < 0.05, **p < 0.01, and ***p < 0.001 by two-way ANOVA with Bonferroni’s post hoc test)

Activation of IRE1α in response to ER stress can induce splicing of XBP1 mRNA. The (ERAI)-Venus Tg mouse was constructed by fusing XBP1 with the fluorescent protein, Venus (Iwawaki et al. 2004). In this system, ER stress is detectable by the expression of Venus owing to a frameshift caused by the splicing of XBP1. To investigate the effects of MeHg on ER stress in the brain, ERAI mice were fed 50 ppm MeHg ad libitum for up to 5 weeks as a model of subchronic MeHg toxicity (Fig. 1a). To clarify the involvement of ER stress in the neurological damage in this model, 4-PBA (Fig. 1b) was administered intraperitoneally at 120 mg/kg/day, a dose sufficient to inhibit rotenone-induced neuronal cell death (Inden et al. 2007). 5 weeks later, immunohistochemical detection of ERAI signals in the somatosensory cortex using GFP antibodies revealed that the ERAI signals induced by MeHg were significantly reduced by concurrent administration of 4-PBA (Fig. 1c, d). Furthermore, a reduction in ERAI signal by 4-PBA was observed in the striatum (Fig. 1e, f).

Analysis of total mercury levels in the brain showed that MeHg administration caused accumulation of total mercury in the somatosensory cortex and striatum of the brain, and that 4-PBA had no effect on brain mercury levels in either of these two regions (Fig. S1a, b). In addition, 4-PBA did not affect the weight loss associated with MeHg administration (Fig. S1c).

### 4-PBA inhibits MeHg-induced activation of the IRE1α-XBP1 pathway

**Fig. 2:**
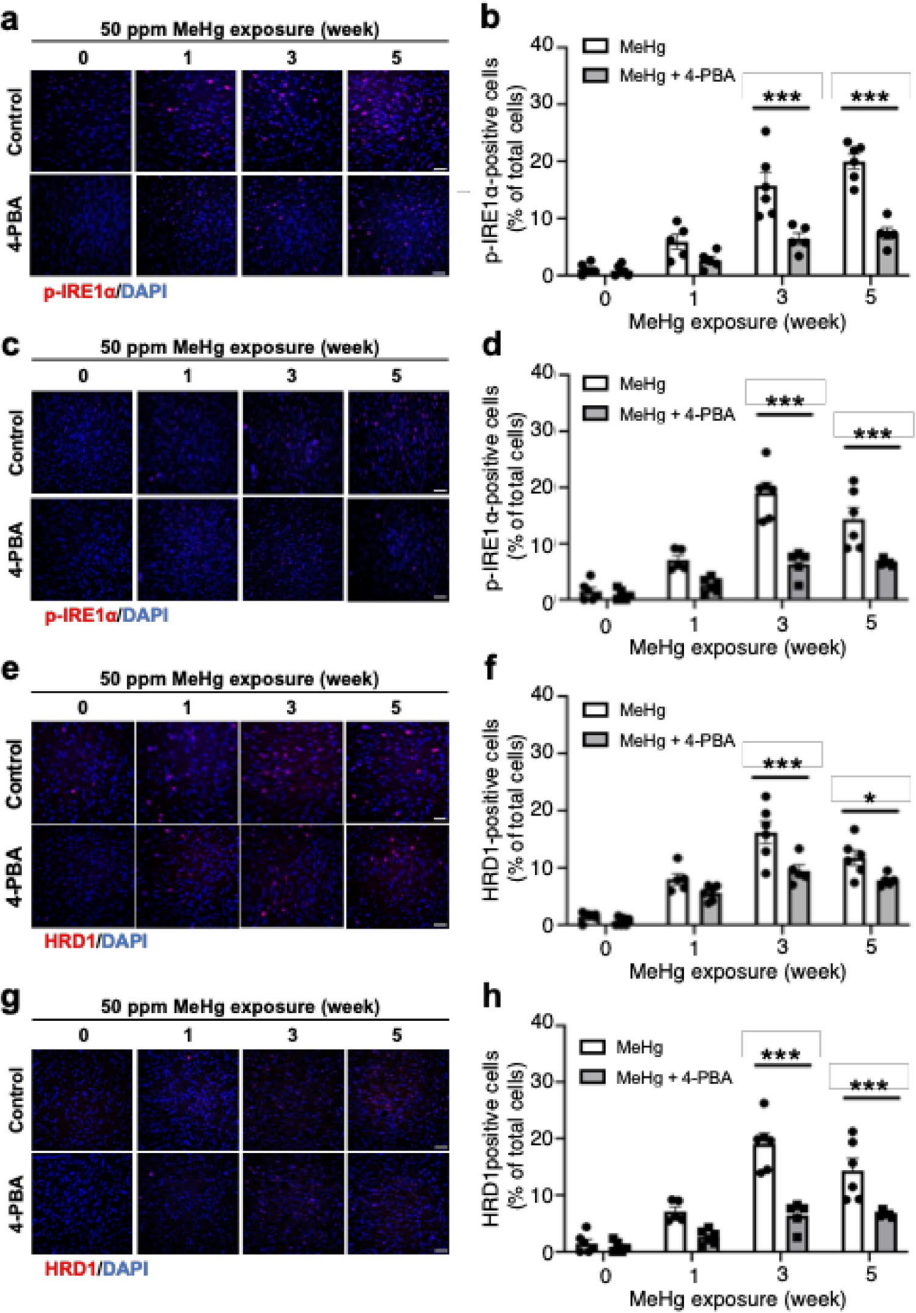
Activation of the IRE1α-XBP1 pathway upon exposure to MeHg was prevented by 4-PBA in vivo. Representative immunostaining for p-IRE1α (**a**, **c**) and HRD1 (**e**, **g**) in the somatosensory cortex and striatum, respectively, of ERAI-transgenic mice. The scale bar represents 50 μm. **b**, **d**, **f**, **h** Quantification of p-IRE1α- and HRD1-positive cells shown in (**a**, **c**, **e**, **g**, respectively). Data are presented as the mean ± s.e.m. (*n* = 5–6, *p < 0.05 and ***p < 0.001 by two-way ANOVA with Bonferroni’s post hoc test)

Next, we analyzed the effects of 4-PBA on IRE1α, an ER stress sensor protein activated by autophosphorylation upon sensing ER stress. Consistent with previous studies (Nomura et al. 2022), MeHg administration enhanced the phosphorylation of IRE1α in a time-dependent manner in the somatosensory cortex (Fig. 2a, b). The activation of IRE1α was inhibited by 4-PBA treatment (Fig. 2a, b). MeHg-induced phosphorylation in the striatum peaked after exposure for 3 weeks, which was suppressed by 4-PBA treatment (Fig. 2c, d). The levels of HMG-CoA reductase 1 (HRD1) were analyzed to examine the activation of XBP1 mRNA splicing by IRE1α. HRD1 is an E3 ubiquitin ligase whose transcription is induced by XBP1 homodimers produced by the splicing of XBP1. MeHg-induced HRD1 levels were analyzed in both cortical somatosensory and striatal regions, reaching a peak 3 weeks after the start of MeHg treatment (Fig. 2e–h). Furthermore, HRD1 expression was reduced by 4-PBA treatment in both regions (Fig. 2e–h).

### 4-PBA inhibits MeHg-induced activation of the PERK pathway

**Fig. 3:**
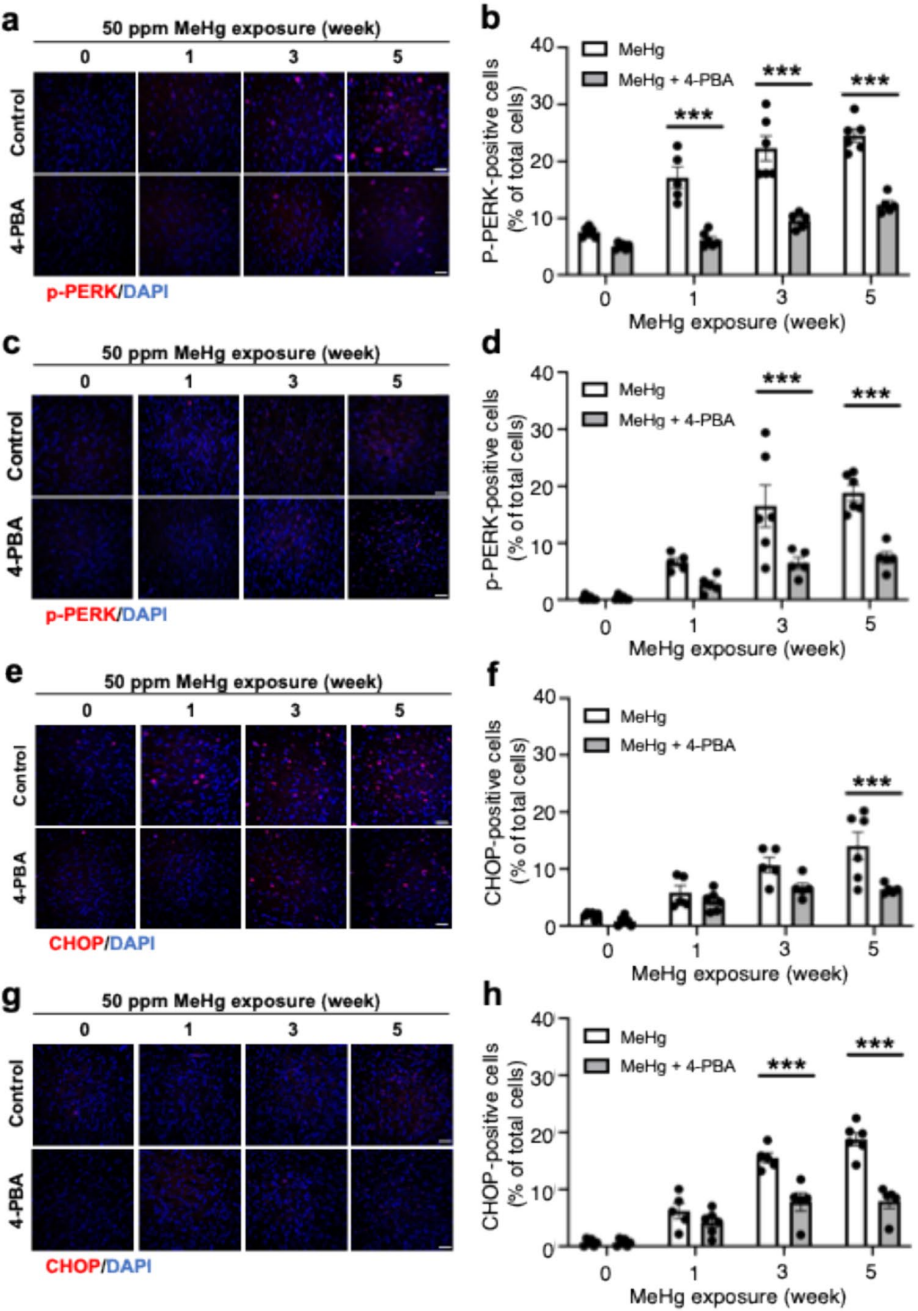
Activation of the PERK pathway upon exposure to MeHg was prevented by 4-PBA in vivo. Representative immunostaining for p-PERK (**a**, **c**) and CHOP (**e**, **g**) in the somatosensory cortex and striatum, respectively, of ERAI-transgenic mice. The scale bar represents 50 μm. **b**, **d**, **f**, **h** Quantification of p-PERK—and CHOP-positive cells shown in (**a**, **c**, **e**, **g**, respectively). Data are presented as the mean ± s.e.m. (*n* = 5–6, ***p < 0.001 by two-way ANOVA with Bonferroni’s post hoc test)

Next, we examined the activity of the PERK pathway, which is involved in the induction of apoptosis in the UPR. PERK is activated by autophosphorylation upon sensing ER stress, and induces the expression of CHOP, a transcription factor associated with the induction of apoptosis. MeHg exposure enhanced PERK phosphorylation, which was suppressed by 4-PBA (Fig. 3a, b). 4-PBA also suppressed MeHg-induced PERK phosphorylation in the striatum (Fig. 3c, d). Subsequent analysis of temporal changes in CHOP levels in the somatosensory cortex and striatum showed that the time-dependent increases in CHOP levels in both regions upon MeHg exposure were suppressed by 4-PBA (Fig. 3e–h).

### 4-PBA inhibits MeHg-induced neuronal cell death

**Fig. 4:**
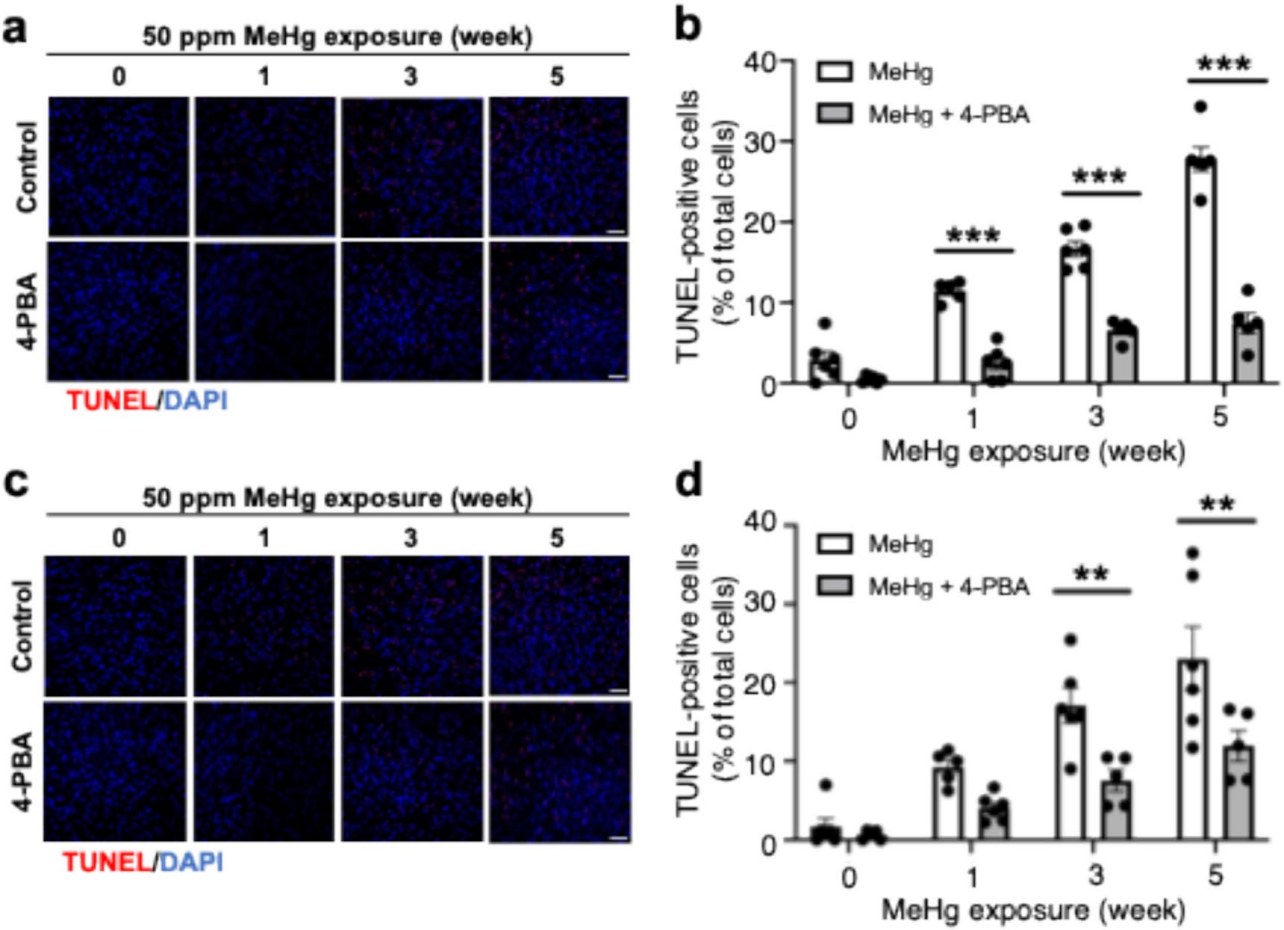
MeHg-induced neuronal cell death is prevented by 4-PBA in vivo. Representative images of TUNEL staining in the somatosensory cortex (**a**) and striatum (**c**) of ERAI-transgenic mice. The scale bar represents 50 μm. **b**, **d** Quantification of apoptotic cells is shown in (**a**, **c**), respectively. The scale bar represents 50 μm. Data are presented as the mean ± s.e.m. (n = 5–6, **p < 0.01, and ***p < 0.001 by two-way ANOVA with Bonferroni’s post hoc test)

As shown in Fig. 3, 4-PBA inhibits activation of the PERK pathway, which is involved in the induction of apoptosis, suggesting that 4-PBA inhibits apoptosis. Therefore, we investigated the effect of 4-PBA on MeHg-induced neuronal apoptosis by analyzing the number of TUNEL-positive cells in TUNEL assays, a marker of apoptosis. 4-PBA significantly inhibited the increase in the number of TUNEL-positive cells in the somatosensory cortex (Fig. 4a, b) and the striatum (Fig. 4c, d).

### 4-PBA ameliorates MeHg-induced neurological dysfunction

**Fig. 5:**
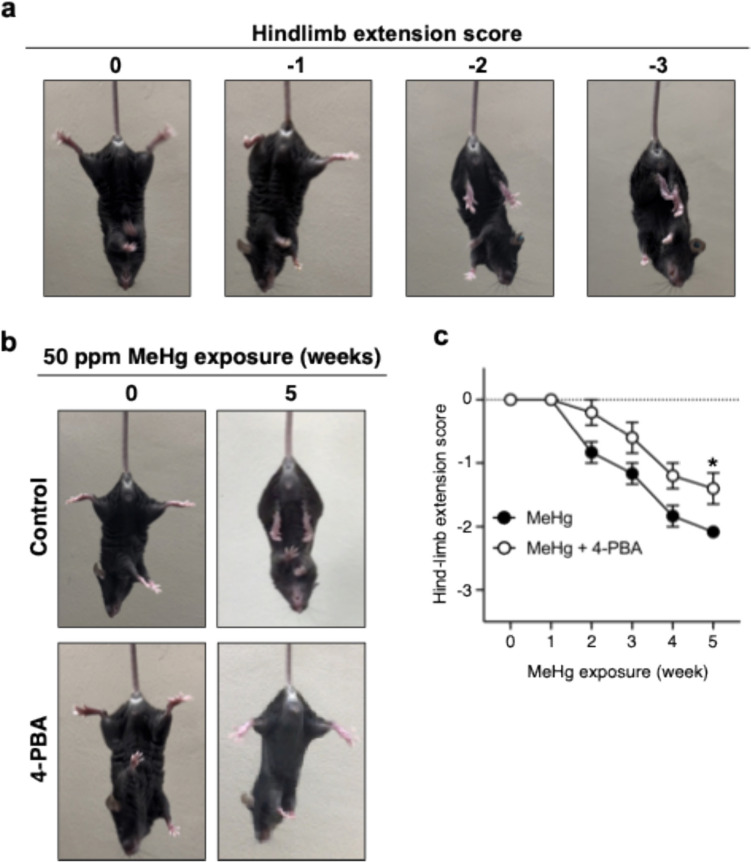
Effect of 4-PBA on MeHg-induced neurological symptoms. **a** Representative images of each score for the hindlimb extension response. normal escape extension = 0, incomplete splay and loss of mobility =  − 1, no splay and loss of mobility =  − 2, complete crossing of the hindlimbs =  − 3. **b** Representative images of mice during the hindlimb clasping test. **c** Quantification of hindlimb splay, which is shown in (**b**). Data are presented as the mean ± s.e.m. (n = 5–6, *p < 0.05 by two-way ANOVA with Bonferroni’s post hoc test)

The 4-PBA suppression of MeHg-induced cell death prompted us to investigate whether 4-PBA could ameliorate the neurological symptoms caused by MeHg exposure. We analyzed the score for hindlimb extension, which is commonly used as a method for analyzing neurological symptoms in a MeHg poisoning model (Iijima et al. 2024a; Weiss et al. 2005). The scoring was analyzed in four stages, as in previous studies (Chakrabarti and Bai 2000; Fujimura et al. 2011). 4-PBA significantly restored MeHg-induced impaired hindlimb extension after the 5th week of treatment (Fig. 5a, b). These results indicate that 4-PBA inhibits MeHg-induced neuronal damage and that the induction of ER stress and changes in UPR activity targeted by 4-PBA are important in the mechanism of MeHg-induced neuronal cell death.

### The post-treatment of 4-PBA

**Fig. 6:**
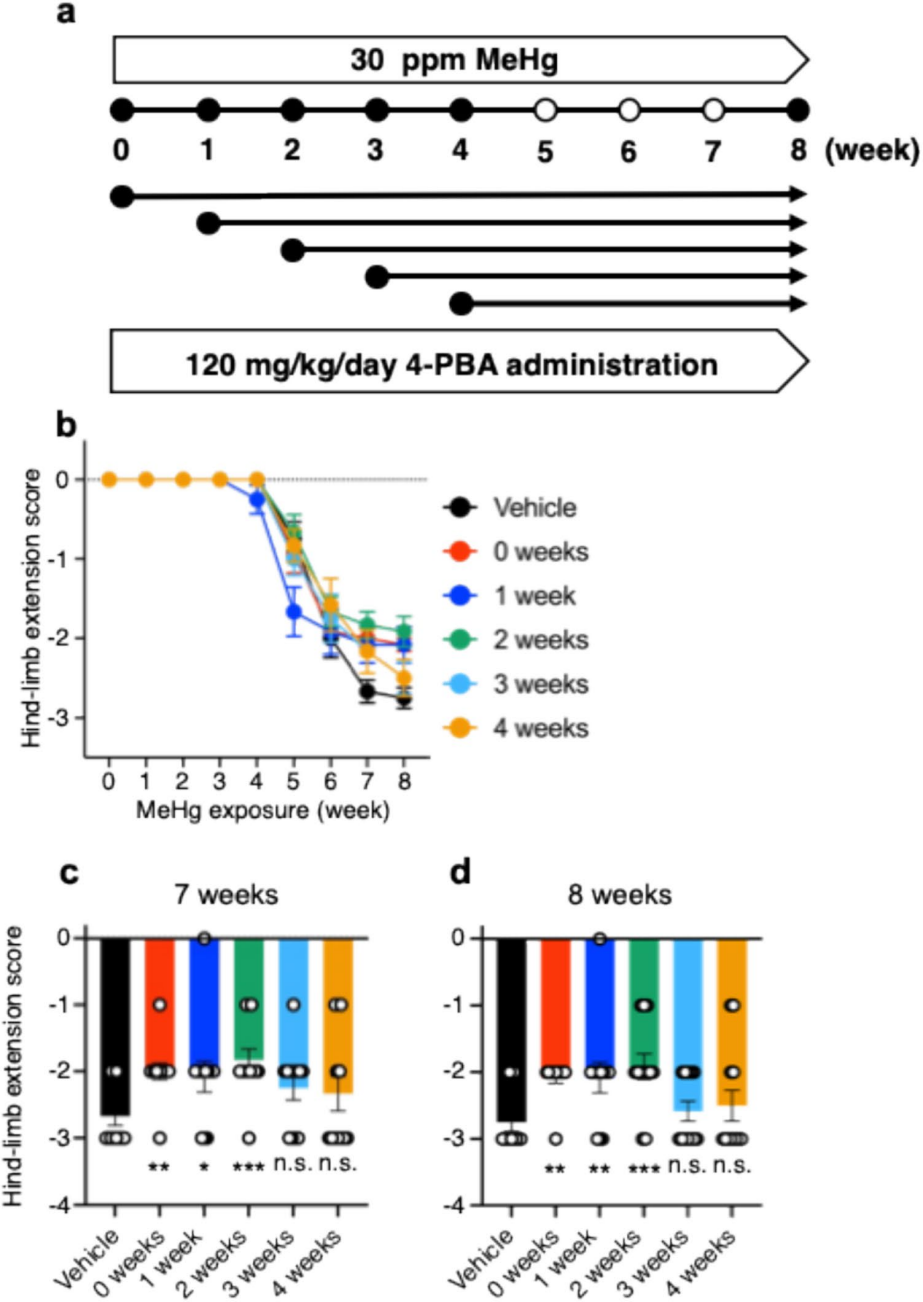
The post-treatment of 4-PBA. **a** Experimental schedule for subacute exposure to MeHg and 4-PBA. WT mice were exposed to 30 ppm MeHg in drinking water for 8 weeks. 4-PBA (120 mg/kg) was administered intraperitoneally once daily. Saline was injected into control animals as a vehicle control. **b** Hindlimb extension responses were measured weekly. Quantification using a 0 to − 3 numerical scale: normal escape extension = 0, incomplete splay and loss of mobility =  − 1, no splay and loss of mobility =  − 2, complete crossing of the hindlimbs =  − 3. **c**, **d** Hindlimb extension scores at 7 and 8 weeks are shown. For mice that died during the period of analysis, measurements up to death were plotted*.* (n = 12, *p < 0.05, **p < 0.01 and ***p < 0.001 by two-way ANOVA with Bonferroni’s post hoc test; n.s., not significant)

We then investigated the progression of disability over time caused by MeHg toxicity. We examined the cut-off point at which irreversible disability occurs and the effective time range between MeHg exposure and the initiation of 4-PBA treatment. We also assessed whether 4-PBA could suppress MeHg toxicity even when there was an interval between the start of MeHg exposure and the beginning of 4-PBA administration. Based on previous studies (Fujimura et al. 2009), WT mice were exposed to 30 ppm MeHg (Fig. 6a). Neurological symptoms and the number of TUNEL-positive cells in the brain were analyzed. Post-treatment of 4-PBA caused temporary weight loss in the 1-week post-treatment group but had no overall effect on weight loss (Fig. S2d). Hindlimb extension measurements showed that suppression of neurological symptoms was also observed when 4-PBA was administered 2 weeks after the start of MeHg exposure (Fig. 6b–d). TUNEL staining was performed to assess neuronal cell death in the somatosensory cortex and striatum. Consistent with the results of the hindlimb extension response, a trend toward suppression of neuronal cell death was observed up to 2 weeks after the start of MeHg exposure (n = 1) (Fig. S2c). These results suggest that the cutoff point for irreversible damage from 30 ppm MeHg poisoning to neurodegeneration is 2 weeks.

## Discussion

The 4-PBA used in this experiment is a chemical chaperone that can stabilize the structure of proteins and reduce ER stress by inhibiting protein aggregation. Intraperitoneal administration of 4-PBA to mice suppresses rotenone-induced neuronal ER stress and cell death (Inden et al. 2007). Furthermore, 4-PBA inhibits UPR activation via MeHg-induced ER stress in vitro (Yang et al. 2022). However, whether 4-PBA inhibits MeHg-induced ER stress in the mouse brain remained unclear. Using ERAI-Venus Tg mice, in which ER stress can be analyzed spatiotemporally, we analyzed ER stress in the somatosensory cortex and striatum, where MeHg-induced neuronal cell death was observed. The results showed that 4-PBA significantly suppressed MeHg-induced increases in the number of ERAI-positive cells (Fig. 1). In contrast, 4-PBA did not affect mercury accumulation in the brain or body weight (Fig. S1). Furthermore, MeHg activation of the IRE1α-XBP1 (Fig. 2) and PERK (Fig. 3) pathways was also inhibited by 4-PBA. We also investigated the effects of 4-PBA on MeHg-induced neuronal cell death using TUNEL staining and found that 4-PBA suppressed the MeHg-induced increase in the number of TUNEL-positive cells (Fig. 4). Importantly, 4-PBA also suppressed the MeHg-induced decrease in hindlimb extension score, which is indicative of neuropathy (Fig. 5). These results demonstrate a preventive effect of 4-PBA on MeHg toxicity. However, MeHg toxicity can cause progressive neurodegeneration and early treatment of this condition is crucial. We therefore analyzed the point at which irreversible damage occurred. The results showed that 4-PBA was effective in reducing neuropathy, even when 4-PBA was started 2 weeks after the start of exposure to 30 ppm MeHg (Fig. 6). These results indicate that inhibition of ER stress is a potential therapeutic strategy against MeHg toxicity in the early phase of exposure to 30 ppm MeHg.

Our findings indicate that ER stress and the UPR, targets of 4-PBA, are the molecular processes that are centrally involved in the development of MeHg toxicity. However, specific factors that directly induce MeHg-induced neuronal cell death remain unclear and studies to demonstrate whether MeHg-induced neuronal cell death can be alleviated in vivo using methods to regulate each factor of the UPR are warranted. Our analysis using CHOP-KO mice suggests that CHOP is not responsible for MeHg-induced neuronal death (Iijima et al. 2024a). Further analysis of PERK pathway involvement in MeHg toxicity should be performed using PERK inhibitors. The ASK1/JNK pathway mediated by IRE1α is a CHOP-independent apoptotic pathway mediated by ER stress. MeHg activates the ASK1/JNK pathway (Fujimura et al. 2009; Usuki et al. 2008), and the effect of this pathway may be one of the reasons why 4-PBA suppressed MeHg-induced cell death in this study. In addition, we did not examine the effect of inhibiting ATF6 pathway activation on MeHg toxicity, which should be addressed in future studies.

Mechanisms other than ER stress have been investigated to determine their involvement in MeHg-induced neuronal cell death. In our study, therapeutic effects were also observed after 2 weeks of post-treatment; therefore, it is expected that although ER stress is the primary mechanism responsible for toxicity during the first two weeks of exposure to 30 ppm MeHg (Fig. 6), cell death may be accelerated by inducing other cell death mechanisms, such as microglial activation.

In addition, MeHg induces oxidative stress-induced neuronal cell death in the mouse brain (Franco et al. 2009). MeHg impairs the intracellular antioxidant system and produces reactive oxygen species (ROS) (Farina et al. 2011; Franco et al. 2009). It also reacts with glutathione (GSH), an intracellular antioxidant, and induces cell death via oxidative stress by depleting GSH (Xu et al. 2012). Additionally, an association between MeHg toxicity and nuclear factor erythroid 2-related factor 2 (NRF2) (Buha et al. 2021), an endogenous antioxidant activator, has been suggested. MeHg can induce ferroptosis via suppression of NRF2 (Xu et al. 2023) and MeHg-induced oxidative stress contributes to ER stress (Zhang and Kaufman 2008). Studies have been conducted to target MeHg-induced oxidative stress by administering chelating agents and antioxidants (Cao et al. 2015; Chang et al. 1978). Chelating agents aim to remove MeHg, whereas antioxidants remove ROS from the blood and brain. However, these approaches do not provide a fundamental solution for MeHg toxicity, and post-MeHg administration treatments targeting oxidative stress did not suppress MeHg toxicity (Fujimura and Usuki 2020). 4-PBA suppresses oxidative stress-induced ER stress, which may also suppress toxicity in post-treatment. It is expected that a combination of 4-PBA and therapeutic agents targeting oxidative stress will effectively inhibit neuronal cell death.

Activation of microglia and release of proinflammatory cytokines may be involved in the mechanism of MeHg-induced neuronal cell death (Hoshi et al. 2019), and activation of the apoptotic pathway mediated by Tumor necrosis factor α (TNFα) is suggested to be involved in MeHg-induced neuronal cell death (Iwai-Shimada et al. 2016). In our previous study using ERAI-Venus Tg mice, we confirmed that the number of activated microglia was increased in the somatosensory cortex of mice treated with 30 ppm MeHg in drinking water (Hiraoka et al. 2021). MeHg-induced neuronal death my therefore involve an additive mechanism of apoptosis induction via the activation of the UPR and microglia.

In this study, the exposure concentration was set to reflect the concentration of MeHg in the brains of Minamata disease patients. In some cases, the total mercury concentration in the brains of Minamata disease patients exceeded 20 ppm (Takeuchi et al. 1962), and we consider that the MeHg dose used in this study does not greatly deviate from this value.

4-PBA did not affect MeHg-induced weight loss (Fig. S1c). This indicates that 4-PBA specifically inhibited neurotoxicity, which indicates that the mechanism of MeHg-induced neuronal cell death may be different from the mechanism in other tissues such as the cardiovascular system (Hong et al. 2012). Further studies are required to understand the mechanisms through which MeHg acts in different tissues. Drugs targeting ER stress have not been used clinically for diseases of the central nervous system. 4-PBA can inhibit rotenone-induced neuronal cell death in a concentration-dependent manner, indicating that 4-PBA is transferred into the brain and exerts an effect (Inden et al. 2007). The present study shows that 4-PBA is a new drug candidate for treating MeHg poisoning. 4-PBA is used to treat chronic urea cycle disorders in children and has been approved by the FDA (Brusilow and Maestri 1996), so it is considered that 4-PBA has sufficient clinical application potential as a drug. However, according to the product information (PDR.net: https://www.pdr.net/drug-summary/?drugLabelId=1782), neurotoxicity symptoms of drowsiness, fatigue, and dizziness have been reported in patients who were administered 4-PBA by intravenous injection (250–300 mg/kg/day for 14 days, with a 4-week interval). This side effect may limit the clinical use of 4-PBA, and it will be necessary to elucidate the mechanism and develop 4-PBA analogs with fewer side effects in the future.

Previous reports have shown that histone deacetylase (HDAC) inhibition by 4-PBA is not associated with the amelioration of ER stress induced by tunicamycin (Carlisle et al. 2021) and that 4-PBA analogs with reduced chaperone activity do not inhibit ER stress-induced neuronal cell death (Mimori et al. 2013). These results strongly suggested that the amelioration of MeHg-induced neuronal cell death was due to 4-PBA chaperone activity. However, it is possible that the HDAC inhibitory effect of 4-PBA is synergistic, and this needs to be further investigated. In conclusion, we examined the importance of ER stress in MeHg toxicity and its potential as a therapeutic target. 4-PBA inhibited ER stress and apoptosis in the mouse brain and suppressed neurological symptoms. These results provide further evidence that ER stress is an important molecular mechanism in MeHg toxicity and new insights into the treatment of MeHg poisoning.

## Supplementary Information

Below is the link to the electronic supplementary material.

Supplementary file1 (DOCX 551 kb)

## Data Availability

The datasets generated and/or analyzed during the current study are available from the corresponding author on reasonable request.

## References

[CR1] Brusilow SW, Maestri NE (1996) Urea cycle disorders: diagnosis, pathophysiology, and therapy. Adv Pediatr 43(1):127–170. 10.1016/S0065-3101(24)00072-0 8794176

[CR2] Buha A, Baralic K, Djukic-Cosic D et al (2021) the role of toxic metals and metalloids in Nrf2 signaling. Antioxidants (Basel) 10(5):630. 10.3390/antiox10050630 33918986 10.3390/antiox10050630 PMC8142989

[CR3] Cao Y, Skaug MA, Andersen O, Aaseth J (2015) Chelation therapy in intoxications with mercury, lead and copper. J Trace Elem Med Biol 31:188–192. 10.1016/j.jtemb.2014.04.010 24894443 10.1016/j.jtemb.2014.04.010

[CR4] Carlisle RE, Farooqi S, Zhang MC et al (2021) Inhibition of histone deacetylation with vorinostat does not prevent tunicamycin-mediated acute kidney injury. PLoS ONE 16(11):e0260519. 10.1371/journal.pone.0260519 34847196 10.1371/journal.pone.0260519 PMC8631648

[CR5] Chakrabarti SK, Bai C (2000) Effects of protein-deficient nutrition during rat pregnancy and development on developmental hindlimb crossing due to methylmercury intoxication. Arch Toxicol 74(4–5):196–202. 10.1007/s002040000112 10959792 10.1007/s002040000112

[CR6] Chang LW, Gilbert M, Sprecher J (1978) Modification of methylmercury neurotoxicity by vitamin E. Environ Res 17(3):356–366. 10.1016/0013-9351(78)90040-3 318524 10.1016/0013-9351(78)90040-3

[CR7] Clarkson TW (1972) The pharmacology of mercury compounds. Annu Rev Pharmacol 12:375–406. 10.1146/annurev.pa.12.040172.002111 4556945 10.1146/annurev.pa.12.040172.002111

[CR8] Compeau GC, Bartha R (1985) Sulfate-reducing bacteria: principal methylators of mercury in anoxic estuarine sediment. Appl Environ Microbiol 50(2):498–502. 10.1128/aem.50.2.498-502.1985 16346866 10.1128/aem.50.2.498-502.1985 PMC238649

[CR9] Eto K, Takeuchi T (1978) A pathological study of prolonged cases of Minamata disease. With particular reference to 83 autopsy cses. Acta Pathol Jpn 28(4):565–584. 10.1111/j.1440-1827.1978.tb00896.x 716883 10.1111/j.1440-1827.1978.tb00896.x

[CR10] Farina M, Aschner M, Rocha JB (2011) Oxidative stress in MeHg-induced neurotoxicity. Toxicol Appl Pharmacol 256(3):405–417. 10.1016/j.taap.2011.05.001 21601588 10.1016/j.taap.2011.05.001 PMC3166649

[CR11] Franco JL, Posser T, Dunkley PR et al (2009) Methylmercury neurotoxicity is associated with inhibition of the antioxidant enzyme glutathione peroxidase. Free Radic Biol Med 47(4):449–457. 10.1016/j.freeradbiomed.2009.05.013 19450679 10.1016/j.freeradbiomed.2009.05.013

[CR12] Fujimura M, Usuki F, Sawada M, Takashima A (2009) Methylmercury induces neuropathological changes with tau hyperphosphorylation mainly through the activation of the c-jun-N-terminal kinase pathway in the cerebral cortex, but not in the hippocampus of the mouse brain. Neurotoxicology 30(6):1000–1007. 10.1016/j.neuro.2009.08.001 19666049 10.1016/j.neuro.2009.08.001

[CR13] Fujimura M, Usuki F, Kawamura M, Izumo S (2011) Inhibition of the Rho/ROCK pathway prevents neuronal degeneration in vitro and in vivo following methylmercury exposure. Toxicol Appl Pharmacol 250(1):1–9. 10.1016/j.taap.2010.09.011 20869980 10.1016/j.taap.2010.09.011

[CR14] Fujimura M, Usuki F (2020) Methylmercury-Mediated Oxidative Stress and Activation of the Cellular Protective System. Antioxidants 9(10):1004. 10.3390/antiox9101004 33081221 10.3390/antiox9101004 PMC7602710

[CR15] Hiraoka H, Nakahara K, Kaneko Y et al (2017) Modulation of Unfolded Protein Response by Methylmercury. Biol Pharm Bull 40(9):1595–1598. 10.1248/bpb.b17-00359 28867746 10.1248/bpb.b17-00359

[CR16] Hiraoka H, Nomura R, Takasugi N et al (2021) Spatiotemporal analysis of the UPR transition induced by methylmercury in the mouse brain. Arch Toxicol 95(4):1241–1250. 10.1007/s00204-021-02982-9 33454823 10.1007/s00204-021-02982-9

[CR17] Hong YS, Kim YM, Lee KE (2012) Methylmercury exposure and health effects. J Prev Med Public Health 45(6):353–363. 10.3961/jpmph.2012.45.6.353 23230465 10.3961/jpmph.2012.45.6.353 PMC3514465

[CR18] Hoshi T, Toyama T, Naganuma A, Hwang G-W (2019) Methylmercury causes neuronal cell death via M1-microglial activation in organotypic slices prepared from mouse cerebral cortex. Fund Toxicol Sci 6(5):167–170. 10.2131/fts.6.167

[CR19] Iijima Y, Miki R, Fujimura M, Oyadomari S, Uehara T (2024a) Methylmercury-induced brain neuronal death in CHOP-knockout mice. J Toxicol Sci 49(2):55–60. 10.2131/jts.49.55 38296529 10.2131/jts.49.55

[CR20] Iijima Y, Miki R, Takasugi N, Fujimura M, Uehara T (2024b) Characterization of pathological changes in the olfactory system of mice exposed to methylmercury. Arch Toxicol 98(4):1163–1175. 10.1007/s00204-024-03682-w 38367039 10.1007/s00204-024-03682-w PMC10944439

[CR21] Inden M, Kitamura Y, Takeuchi H et al (2007) Neurodegeneration of mouse nigrostriatal dopaminergic system induced by repeated oral administration of rotenone is prevented by 4-phenylbutyrate, a chemical chaperone. J Neurochem 101(6):1491–1504. 10.1111/j.1471-4159.2006.04440.x 17459145 10.1111/j.1471-4159.2006.04440.x

[CR22] Iwai-Shimada M, Takahashi T, Kim MS et al (2016) Methylmercury induces the expression of TNF-alpha selectively in the brain of mice. Sci Rep 6(1):38294. 10.1038/srep38294 27910896 10.1038/srep38294 PMC5133575

[CR23] Iwawaki T, Akai R, Kohno K, Miura M (2004) A transgenic mouse model for monitoring endoplasmic reticulum stress. Nat Med 10(1):98–102. 10.1038/nm970 14702639 10.1038/nm970

[CR24] Kerper LE, Ballatori N, Clarkson TW (1992) Methylmercury transport across the blood-brain barrier by an amino acid carrier. Am J Physiol 262(5 Pt 2):R761–R765. 10.1152/ajpregu.1992.262.5.R761 1590471 10.1152/ajpregu.1992.262.5.R761

[CR25] King JK, Kostka JE, Frischer ME, Saunders FM (2000) Sulfate-reducing bacteria methylate mercury at variable rates in pure culture and in marine sediments. Appl Environ Microbiol 66(6):2430–2437. 10.1128/AEM.66.6.2430-2437.2000 10831421 10.1128/aem.66.6.2430-2437.2000 PMC110551

[CR26] Kubota K, Niinuma Y, Kaneko M et al (2006) Suppressive effects of 4-phenylbutyrate on the aggregation of Pael receptors and endoplasmic reticulum stress. J Neurochem 97(5):1259–1268. 10.1111/j.1471-4159.2006.03782.x 16539653 10.1111/j.1471-4159.2006.03782.x

[CR27] Mahaffey KR, Clickner RP, Bodurow CC (2004) Blood organic mercury and dietary mercury intake: National Health and Nutrition Examination Survey, 1999 and 2000. Environ Health Perspect 112(5):562–570. 10.1289/ehp.6587 15064162 10.1289/ehp.6587 PMC1241922

[CR28] Makino K, Okuda K, Sugino E et al (2015) Correlation between attenuation of protein disulfide isomerase activity through S-mercuration and neurotoxicity induced by methylmercury. Neurotox Res 27(2):99–105. 10.1007/s12640-014-9494-8 25288108 10.1007/s12640-014-9494-8

[CR29] Mimori S, Ohtaka H, Koshikawa Y et al (2013) 4-Phenylbutyric acid protects against neuronal cell death by primarily acting as a chemical chaperone rather than histone deacetylase inhibitor. Bioorg Med Chem Lett 23(21):6015–6018. 10.1016/j.bmcl.2013.08.001 24044874 10.1016/j.bmcl.2013.08.001

[CR30] Nomura R, Takasugi N, Hiraoka H et al (2022) Alterations in UPR signaling by methylmercury trigger neuronal cell death in the mouse brain. Int J Mol Sci 23(23):15412. 10.3390/ijms232315412 36499738 10.3390/ijms232315412 PMC9738736

[CR31] Ricobaraza A, Cuadrado-Tejedor M, Perez-Mediavilla A, Frechilla D, Del Rio J, Garcia-Osta A (2009) Phenylbutyrate ameliorates cognitive deficit and reduces tau pathology in an Alzheimer’s disease mouse model. Neuropsychopharmacology 34(7):1721–1732. 10.1038/npp.2008.229 19145227 10.1038/npp.2008.229

[CR32] Takeuchi T, Morikawa N, Matsumoto H, Shiraishi Y (1962) A pathological study of Minamata disease in Japan. Acta Neuropathol 2(1):40–57. 10.1007/bf00685743

[CR33] Usuki F, Fujita E, Sasagawa N (2008) Methylmercury activates ASK1/JNK signaling pathways, leading to apoptosis due to both mitochondria- and endoplasmic reticulum (ER)-generated processes in myogenic cell lines. Neurotoxicology 29(1):22–30. 10.1016/j.neuro.2007.08.011 17920127 10.1016/j.neuro.2007.08.011

[CR34] Walter P, Ron D (2011) The unfolded protein response: from stress pathway to homeostatic regulation. Science 334(6059):1081–1086. 10.1126/science.1209038 22116877 10.1126/science.1209038

[CR35] Weiss B, Stern S, Cox C, Balys M (2005) Perinatal and lifetime exposure to methylmercury in the mouse: behavioral effects. Neurotoxicology 26(4):675–690. 10.1016/j.neuro.2005.05.003 15970329 10.1016/j.neuro.2005.05.003

[CR36] Xu B, Xu ZF, Deng Y, Liu W, Yang HB, Wei YG (2012) Protective effects of MK-801 on methylmercury-induced neuronal injury in rat cerebral cortex: involvement of oxidative stress and glutamate metabolism dysfunction. Toxicology 300(3):112–120. 10.1016/j.tox.2012.06.006 22722016 10.1016/j.tox.2012.06.006

[CR37] Xu X, Wang SS, Zhang L et al (2023) Methylmercury induced ferroptosis by interference of iron homeostasis and glutathione metabolism in CTX cells. Environ Pollut 335:122278. 10.1016/j.envpol.2023.122278 37517642 10.1016/j.envpol.2023.122278

[CR38] Yang C-Y, Liu S-H, Su C-C, et al (2022) Methylmercury Induces Mitochondria- and Endoplasmic Reticulum Stress-Dependent Pancreatic β-Cell Apoptosis via an Oxidative Stress-Mediated JNK Signaling Pathway. Int J Mol Sci 23(5):2858. 10.3390/ijms23052858 35270009 10.3390/ijms23052858 PMC8910963

[CR39] Zhang K, Kaufman RJ (2008) From endoplasmic-reticulum stress to the inflammatory response. Nature 454(7203):455–462. 10.1038/nature07203 18650916 10.1038/nature07203 PMC2727659

