# Supplementary material for: Therapeutic potential of 4-phenylbutyric acid against methylmercury-induced neuronal cell death in mice
Source: Arch Toxicol. 2024 Oct 27;99(2):563–74. doi: 10.1007/s00204-024-03902-3 (PMC11775073; doi:10.1007/s00204-024-03902-3)
Supplement: Supplementary file 1 — Supplementary file1 (DOCX 551 kb) [file 204_2024_3902_MOESM1_ESM.docx]

**Fig. S1** Effect of 4-PBA on mercury accumulation in the brain. Quantification of total mercury concentration in the cerebral cortex (**a**) and striatum (**b**) of ERAI-transgenic mice exposed to MeHg for the indicated times. Data are the mean ± s.e.m. (*n* = 5–6). No significant difference was observed between vehicle and 4-PBA groups. **(c)** Body weights of wild-type (WT) and 4-PBA-treated mice. These mice received 0 ppm (vehicle) or 30 ppm MeHg from drinking water for 8 weeks. For mice that died during the period of analysis, measurements up to death were plotted. Data are presented as the mean ± s.e.m. (*n* = 5–6, by two-way ANOVA with Bonferroni’s *post hoc* test)

**Fig. S2** 4-PBA post-treatment does not affect weight loss in mice. **a, b** Hindlimb extension scores at 5 and 6 weeks were plotted. For mice that died during the period of analysis, measurements up to death were plotted. **c** Mice were euthanized at 8 weeks after the start of MeHg administration and brain tissue was analyzed by immunostaining. Representative images of TUNEL staining in the somatosensory cortex (Cr) and striatum (St) of 4-PBA-treated WT mice exposed to MeHg (n = 1). **d** Body weights of 4-PBA-treated mice exposed to 30 ppm MeHg. For mice that died during the period of analysis, measurements up to death were plotted. Data are presented as the mean ± s.e.m. (*n* = 12, *** *p* < 0.001 by two-way ANOVA with Bonferroni’s *post hoc* test; n.s., not significant)
